# Effect of serum inflammatory factors in predicting co‐infection with influenza viruses and Omicron

**DOI:** 10.1002/iid3.1158

**Published:** 2024-01-22

**Authors:** Chengli Zhang, Wei Wang, Xianglin Luo, Minggang Yin, Xiaolong Guo

**Affiliations:** ^1^ Zigong First People's Hospital Zigong Academy of Medical Sciences Zigong China; ^2^ Information Department Zigong First People's Hospital Zigong China; ^3^ Xinqiao Hospital Army Medical University Chongqing China

**Keywords:** C‐reactive protein, influenza viruses, Omicron, prealbumin, predicting co‐infection

## Abstract

**Objectives:**

To identify the key differences in laboratory indicators between mono‐infection and co‐infection by influenza viruses and Omicron to facilitate timely adjustments in patient treatment strategies.

**Methods:**

Prealbumin and C‐reactive protein (CRP) levels were analyzed in 161 COVID‐19 cases infected by SARS‐CoV‐2 (wild type), 299 cases infected by Omicron, 95 cases infected by influenza virus A/B (Flu A/B) and 133 co‐infection cases infected with Flu A/B and Omicron. The receiver operating characteristic (ROC) curve and logistic regression equation were used to analyze the clinical predictive capacity of prealbumin and CRP in coinfected patients.

**Results:**

The co‐infected and wild‐type infected patients had significantly different CRP and prealbumin levels compared to mono‐infected patients with Omicron or Flu A/B (*p* < .001). The ROC curve results indicated that prealbumin was more efficient than CRP in identifying co‐infection from Omicron (AUC: 0.867 vs. 0.724) or Flu A/B (AUC: 0.797 vs. 0.730), and joint prediction significantly improved the diagnostic ability to discriminate co‐infection from mono‐infection (AUC: 0.934 and 0.887).

**Conclusion:**

The findings suggest that prealbumin is a valuable indicator that can warn of co‐infection and guide timely treatment decisions. Joint prediction may offer an even more effective diagnostic tool for discriminating co‐infection from mono‐infection.

1

The wave of COVID‐19 epidemics is subsiding as the world moves into the postepidemic era, but the virus is not going away. New variants of severe acute respiratory syndrome coronavirus 2 (SARS‐CoV‐2) are emerging. Although the new variants of SARS‐CoV‐2 are becoming less pathogenic,^1^ they appear to be transmitted more rapidly and are still capable of causing widespread epidemics.^2^ Influenza viruses can also cause epidemics occasionally.^3^ Nowadays, influenza viruses cause a large number of epidemic diseases. SARS‐CoV‐2 is similar to influenza viruses in terms of transmission routes and seasonal epidemics, and co‐infection with both viruses is possible.^4^ Recently, a study of influenza viruses co‐infection with SARS‐CoV‐2 was reported in the Lancet, and the clinical outcome showed that co‐infection with influenza viruses and Omicron significantly increased the likelihood of receiving invasive mechanical ventilation and in‐hospital mortality.^5^ Dual infection with both viruses may increase the extent and severity of virus‐induced lung damage. Therefore, it is crucial to identify the key difference in laboratory indicators in mono‐infection or co‐infection with influenza viruses and Omicron. This study aims to address this issue. By recognizing these differences, healthcare providers may develop more effective treatment strategies, especially in cases involving co‐infection, which may potentially exacerbate lung damage caused by both viruses.

Previously, we found that serum prealbumin decreased markedly in patients with SARS‐CoV‐2 (wild type) infection, and meaningful for early triage of SARS‐CoV‐2 (wild type) infection.^6^ Since then, our findings have been successively verified by many other studies.^7^ Prealbumin is mainly derived from the liver, which could be consumed as a substrate for inflammatory repair in acute or chronic diseases.^8^, ^9^ Therefore, the prealbumin may be used as a key laboratory indicator to identify the mono‐infection or co‐infection with Omicron and influenza viruses.

Here, we conducted in 161 COVID‐19 cases infected by SARS‐CoV‐2 (wild type), 299 COVID‐19 cases infected by Omicron, 95 cases infected by influenza virus A/B (Flu A/B) and 133 co‐infection cases infected with Flu A/B and Omicron. Table 1 showed the baseline characteristics of patients in four groups. The levels of prealbumin and C‐reactive protein (CRP) had been analyzed and compared with each other (Table S1). Serum prealbumin was significantly higher in the Omicron group than wild‐type group, and CRP was lower in the Omicron group than wild‐type group (Figure 1A,B). This was mainly due to the attenuation of pathogenicity with a lower inflammatory response in Omicron infections than in wild‐type.^10^ The other reason could be that the vaccine provides some protection to the patients, which is consistent with existing research.^11^ None of the wild‐type infected patients received the COVID‐19 vaccine, while over 90% of the Omicron‐infected patients received at least one dose of the SARS‐CoV‐2 vaccine. The changes of serum CRP and prealbumin had no definitely significance between Omicron and Flu A/B groups (Figure 1A,B and Table S1). Whereas the serum CRP and prealbumin were significantly changed in co‐infection group compared with Omicron or Flu A/B mono‐infection group (Figure 1A,B).

**Table 1:** Baseline characteristics of patients in different groups.

	Wild type^a^ group (*n* = 161)	Mono‐infection group		Co‐infection group (n = 133)	*P* value
		Omicron group (*n* = 299)	Flu A/B Group (*n* = 95)		
**Age, years**					
Mean (SD)	46.76 (14.90)	44.94 (14.14)	48.45 (22.93)	58.12 (17.23)	.181
≤39	53 (32.92%)	105 (35.12%)	27 (28.42%)	38 (28.57%)	
40–59	74 (45.96%)	144 (48.16%)	46 (48.42%)	57 (42.86%)	
≥60	34 (21.12%)	50 (16.72%)	22 (23.16%)	38 (28.57%)	
**Sex**					
Female	82 (50.93%)	116 (38.80%)	45 (47.37%)	58 (43.61%)	.076
Male	79 (49.07%)	183 (61.20%)	50 (52.63%)	75 (56.39%)	
**Common symptoms**					
Fever	101/161	171/299	81/95	127/133	<.001
Cough	107/161	191/299	59/95	119/133	<.001
**Laboratory findings**					
CRP, CRP, median (IQR) (0–5 mg/L)	8.76 (2.94–26.0)	3.20 (1.20–10.30)	3.3 (1.09–11.30)	18.0 (3.15–54.42)	<.001
>5 mg/L	98/161 (60.87%)	127/299 (42.47%)	37/95 (38.95%)	93/133 (69.92%)	
PA, median (IQR) (180‐400 mg/L)	161 (125.5–197)	252 (217.0–292.0)	231 (202.0–255.0)	149 (108–204.50)	<.001
<180 mg/L	102/161 (63.35%)	27/299 (9.03%)	16/95 (16.84%)	91/133 (68.42%)	

^a^ Wild type is an abbreviation for SARS‐COV‐2 (wild type). *p* < .01 was considered statistically significant.

**Figure 1:**
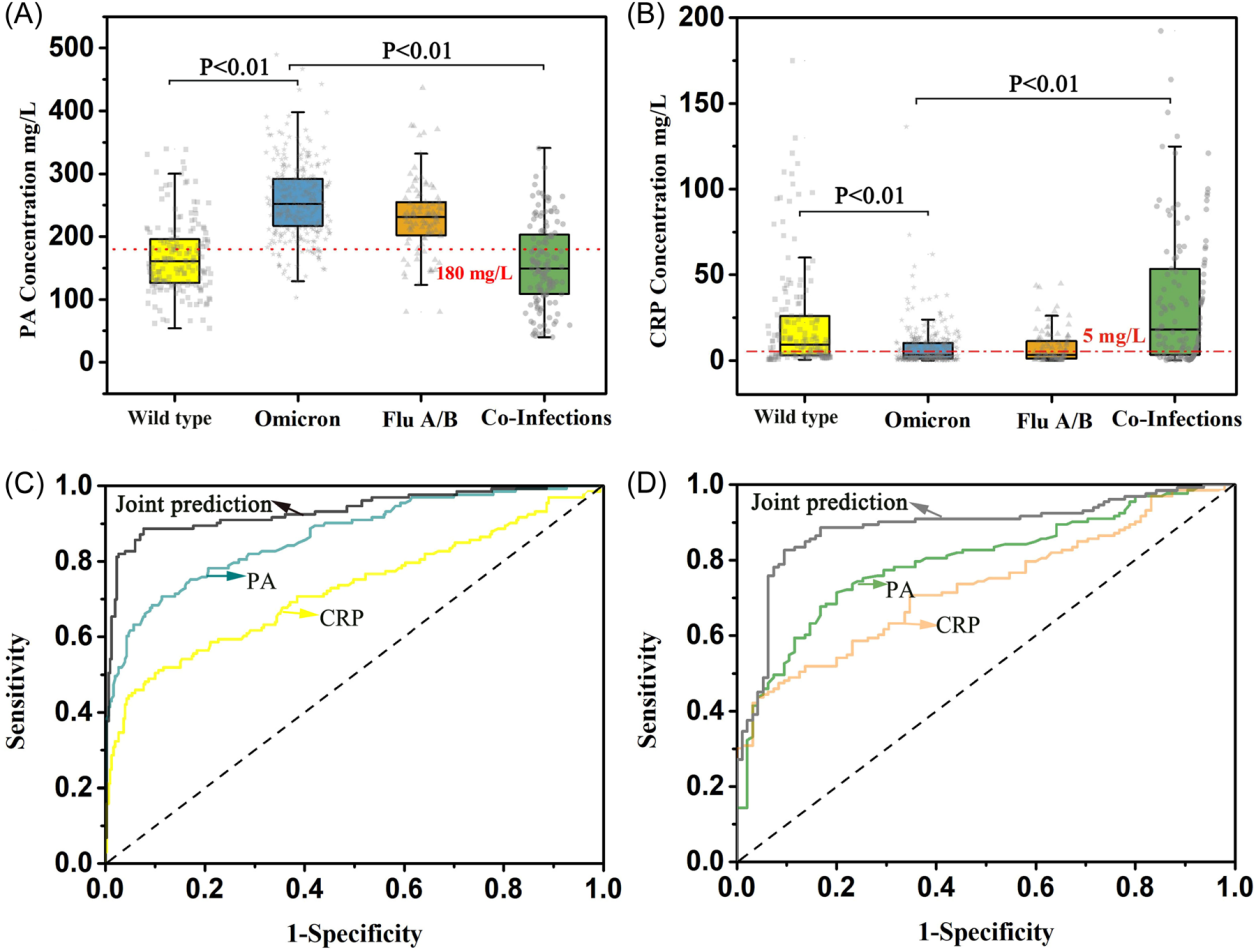
Joint serum prealbumin and CRP improve the ability to discriminate co‐infection from Omicron or influenza virus mono‐infection. The distribution of serum prealbumin and CRP in different groups and the ROC curve for distinguish of co‐infection. (A and B) The concentration of serum prealbumin and CRP in SARS‐Cov‐2 (Wild type) group, Omicron group, Flu A/B group, and co‐infection group with Omicron and Flu A/B. (C) The ROC results of prealbumin and CRP in Omicron and Flu A/B co‐infection group compared to Omicron mono‐infection. (D) The ROC results of prealbumin and CRP in Omicron and Flu A/B co‐infection group compared to Flu A/B mono‐infection.

The ROC curve was used to analyze the clinical predictive value of prealbumin and CRP in coinfected patients (Figure 1C,D). For Omicron versus co‐infection group, the prealbumin with an area under the ROC curve (AUC) of 0.867 at the optimal cut‐off value of 191.5 mg/L, a sensitivity of 0.707, and a specificity of 0.886 (Table S2). For Flu A/B versus co‐infection group, the prealbumin with an AUC of 0.797 at the optimal cut‐off value of 196.4 mg/L, a sensitivity of 0.714, and a specificity of 0.800 (Table S2). In a similar way, the ROC results of the CRP also had a significant in‐diagnostic effect，please see the Figure 1C,D and Table S2. The results indicated that prealbumin was more efficient than CRP in identifying co‐infection from Omicron or Flu A/B mono‐infection (Supplementary Data). What's more, joint the serum prealbumin and CRP to establish a logistic regression equation has a better diagnostic significance for Omicron and Flu A/B co‐infection (Omicron vs. co‐infection: AUC = 0.934; Flu A/B vs. co‐infection: AUC = 0.887) (Figure 1C,D and Table S2). For a detailed description, please see the Supplementary Data. Our results suggested that joint prediction can significantly improve the ability to discriminate co‐infection from mono‐infection.

Significant changes in prealbumin and CRP were observed in coinfected patients, and these indicators could serve as valuable tools for the early identification of co‐infection alongside epidemiology and common symptoms. Prealbumin was superior to CRP in identifying co‐infection. In particular, joint prediction can significantly improve the ability to discriminate co‐infection from mono‐infection. The serum CRP and prealbumin are generally and routinely detected in patients with respiratory infections. CRP and prealbumin serve as an early warning of co‐infection, which can help healthcare workers assess whether co‐infection is present, and alert healthcare workers to perform nucleic acid testing to identify the pathogens and adjust treatment strategy timely. Attention to serum CRP and prealbumin levels can help physicians make timely decisions and reduce the risk of invasive ventilation and in‐hospital mortality of patients. We found that prealbumin and CRP still has a distinguishing effect on Omicron and Flu A/B co‐infection compared to the mono‐infection.

Further research is required to investigate the role of serum inflammatory factors in indicating co‐infection during routine respiratory viral infections.

## AUTHOR CONTRIBUTIONS

**Chengli Zhang**: Data curation; formal analysis; investigation; methodology; project administration; writing—original draft; writing—review & editing. **Wei Wang**: Conceptualization; data curation; formal analysis; software. **Xianglin Luo**: Data curation. **Minggang Yin**: Project administration; resources. **Xiaolong Guo**: Data curation; methodology; validation; visualization; writing—original draft; writing—review & editing.

## CONFLICT OF INTEREST STATEMENT

The authors declare no conflict of interest.

## ETHICS STATEMENT

The study was approved by the Ethics Committee of Zigong First People's Hospital and Chongqing Public Health Medical Center, Southwest University Public Health Hospital.

## Supporting information

Supporting information.

## Data Availability

Data available on request from the authors.
