# Supplementary material for: Effect of serum inflammatory factors in predicting co‐infection with influenza viruses and Omicron
Source: Immun Inflamm Dis. 2024 Jan 22;12(1):e1158. doi: 10.1002/iid3.1158 (PMC10802130; doi:10.1002/iid3.1158)
Supplement: Supplementary file 1 — Supporting information. [file IID3-12-e1158-s001.docx]

**Supplementary Data**

**1. Methods**

**1.1 Patients and data collection**

During the period from Dec. 2019 to Feb. 2023, 688 patients were included in the analysis. 161 patients were diagnosed with SARS-CoV-2（Wild type) infection in Chongqing, China. All of them were included in the Wild type group and none of whom received COVID-19 vaccine. 299 patients were diagnosed with Omicron infection in Zigong, China, all of them were included to Omicron group. 95 patients were diagnosed with influenza A/B virus infection in Zigong, China, all of them were included to Flu A/B group. 133 patients were diagnosed with co-infection by Flu A/B and Omicron in Zigong, China. All of them were included to co-infection group. More than 90% of patients of in these three groups received COVID-19 vaccine[1]. All patients had a primary infection with the SARS-CoV-2. According to inclusion and exclusion criteria, all patients included in this study had no major underlying diseases in the past. The COVID-19 were diagnosed based on criteria issued by the National Health Commission of China. The patients with various cancer, COPD, liver diseases or kidney diseases or other typical metabolic diseases are excluded from our study. Children and pregnant women are excluded from our study. The laboratory data of serum prealbumin and CRP at initial consultation for enrolled patients had been extracted and used for analysis. The CRP and prealbumin were assayed by immunoturbidimetric assay. The study was approved by the Ethics Committee of Zigong First People’s Hospital and Chongqing Public Health Medical Center, Southwest University Public Health Hospital (Ethic (M)2023-017).

**1.2 Statistical analysis**

We present continuous measurements as mean (SD) if they are normally distributed or median (IQR) if they are not. Categorical variables were described as frequency rates and percentages. For the continuous variables, t-test was employed to analyze the difference. If the variances were not normally distributed, the Mann-Whitney U test was used for the two-group analysis and the Kruskal-Wallis H-test was used for the multi-group analysis. Proportions for categorical variables were compared using the χ^2^ test. Receiver operator curves (ROC) to assess the predictive efficacy of CRP and prealbumin on Omicron or Flu A/B monoinfection vs. co-infection. Logistic regression analysis combined with ROC curves were used to evaluate the multiparameter diagnostic efficacy for the joint prediction. All statistical analyses were performed using the SPSS 20.0 (SPSS Inc., Chicago, IL, USA) software package. A two tailed P value of <0.01 was considered statistically significant.

**2. Result**

**2.1 Logistic regression analysis for joint prediction**

By logistic regression analysis, CRP and prealbumin had the ability to predict co-infection independently (P<0.01). A joint prediction model was further developed by logistic regression of the obtained parameters (CRP, prealbumin). In this study, a new variable was introduced, named the joint variable P. Logit(P1) = 4.835 - 0.033*prealbumin + 0.069*CRP (Omicron vs. co-infection) and Logit( P2) = 3.763 - 0.023*prealbumin + 0.061*CRP ( Flu A/B vs. co-infection). Then, the P parameters, CRP and prealbumin were jointly evaluated by ROC curves for multiparametric diagnostic efficacy (Figure 1 C&D). The results showed that the AUC of the joint variable was greater than that these single parameters (Table S2). This indicated that the joint variable was more valuable than single parameters for predicting co-infection, probably because the logistic regression model combined information from both CRP and prealbumin to reflect the characteristics of co-infection from different perspectives, thus suggesting that combining the two is more valuable for predicting co-infection.

| **Table S1. Results of Kruskal-Wallis H test for serum prealbumin and CRP in four groups of patients.** | | | | |
| --- | --- | --- | --- | --- |
| **Laboratory findings** | **CRP** | | **Prealbumin** | |
|  | P Value | Adj. P Value ^a^ | P Value | Adj. P Value ^a^ |
| **Flu A/B group vs. Omicron group**  **Group** | 0.835 | 1.000 | 0.003 | 0.017 |
| **Flu A/B group** **vs. Wild type group** | 0.000 | 0.000 | 0.000 | 0.000 |
| **Flu A/B group vs. co-infection group** | 0.000 | 0.000 | 0.000 | 0.000 |
| **Omicron group vs. Wild type group** | 0.000 | 0.000 | 0.000 | 0.000 |
| **Omicron group vs. co-infection group** | 0.000 | 0.000 | 0.000 | 0.000 |
| **Wild type group vs. co-infection group** | 0.088 | 0.527 | 0.381 | 1.000 |
| ^a^ Significance values have been adjusted for multiple testing by Bonferroni correction.  Wild type is an abbreviation for SARS-CoV-2 (wild type). | | | | |

| **Table S2. ROC curve results of prealbumin and CRP for co-infection.** | | | | | | |
| --- | --- | --- | --- | --- | --- | --- |
|  | Laboratory findings | Cut-off value | AUC | Sensitivity | Specificity | P Value |
| **Omicron vs.**  **co-infection** | Prealbumin | 191.5 mg/L | 0.867 | 0.707 | 0.886 | <0.001 |
|  | CRP | 16.84 mg/L | 0.724 | 0.511 | 0.893 | <0.001 |
|  | Joint variable P | 0.33 | 0.934 | 0.887 | 0.923 | <0.001 |
| **Flu A/B vs.**  **co-infection** | Prealbumin | 196.40 mg/L | 0.797 | 0.714 | 0.800 | <0.001 |
|  | CRP | 30.145 mg/L | 0.730 | 0.436 | 0.958 | <0.001 |
|  | Joint variable P | 0.66 | 0.887 | 0.827 | 0.905 | <0.001 |

**References**

[1] Gewei Wang, Hui Wang, Wenjin Wang, Yafeng Wang, Xinxin Chen, Jinquan Gong, Qinqin Meng, Yaohui Zhao, Determinants of COVID-19 vaccination status and hesitancy among older adults in China, nature medicine. 29 (2023) 623-31. <https://doi.org/https://doi.org/10.1038/s41591-023-02241-7>.
